# Growth in children with chronic kidney disease and associated risk factors for short stature

**DOI:** 10.1590/2175-8239-JBN-2023-0203en

**Published:** 2024-07-29

**Authors:** Virgínia Barbosa de Melo, Danielle Barbosa da Silva, Matheus Dantas Soeiro, Lucas Cavalcante Tenório de Albuquerque, Henderson Edward Firmino Cavalcanti, Marcela Correa Araújo Pandolfi, Rosilene Mota Elias, Rosa Maria Affonso Moysés, Emília Maria Dantas Soeiro

**Affiliations:** 1 Instituto de Medicina Integral Professor Fernando Figueira, Recife, PE, Brazil.; 2 Faculdade Pernambucana de Saúde, Recife, PE, Brazil.; 3 Universidade de São Paulo, Hospital das Clínicas, São Paulo, SP, Brazil.; 4 Universidade de São Paulo, Faculdade de Medicina, Laboratório de Investigação Médica, São Paulo, SP, Brazil.; 5 Universidade Federal de Pernambuco, Faculdade de Medicina do Recife, Recife, PE, Brazil.

**Keywords:** Renal Insufficiency, Chronic, Failure to Thrive, Chronic Kidney Disease-Mineral and Bone Disorder, Growth, Child Nutrition

## Abstract

**Introduction:**

Growth failure in chronic kidney disease is related to high morbidity and mortality. Growth retardation in this disease is multifactorial. Knowing the modifiable factors and establishing strategies to improve care for affected children is paramount.

**Objectives:**

To describe growth patterns in children with chronic kidney disease and the risk factors associated with short stature.

**Methods:**

We retrospectively analyzed anthropometric and epidemiological data, birth weight, prematurity, and bicarbonate, hemoglobin, calcium, phosphate, alkaline phosphatase, and parathormone levels of children with stages 3–5 CKD not on dialysis, followed for at least one year.

**Results:**

We included 43 children, the majority of which were boys (65%). The mean height/length /age z-score of the children at the beginning and follow-up was –1.89 ± 1.84 and –2.4 ± 1.67, respectively (p = 0.011). Fifty-one percent of the children had short stature, and these children were younger than those with adequate stature (p = 0.027). PTH levels at the beginning of the follow-up correlated with height/length/age z-score. A sub-analysis with children under five (n = 17) showed that 10 (58.8%) of them failed to thrive and had a lower weight/age z-score (0.031) and lower BMI/age z-score (p = 0.047).

**Conclusion:**

Children, particularly younger ones, with chronic kidney disease who were not on dialysis had a high prevalence of short stature. PTH levels were correlated with height z-score, and growth failure was associated with worse nutritional status. Therefore, it is essential to monitor the growth of these children, control hyperparathyroidism, and provide nutritional support.

## Introduction

Growth retardation in the context of chronic kidney disease (CKD) causes multiple unfavorable outcomes, including low quality of life, low self-esteem, worse school performance, and high morbidity and mortality^1^. The changes in insulin-like growth factor-binding proteins (IGFBPs) in children with CKD lead to a reduced IGF activity in the chondrocytes of the growth plate. This mechanism occurs due to the competition with type 1 IGF receptors and helps to explain the resistance to growth hormone (GH)^2^.

Factors associated with CKD, such as nutritional deficiency, metabolic acidosis, anemia, and mineral bone disease, aggravate the condition in these children. The dietary restrictions imposed by CKD treatment and the reduced intake secondary to the disease contribute to nutritional deficiency^1^. Metabolic acidosis is a negative stimulus on GH secretion^1^. Anemia harms cellular oxygenation and affects growth^3^. Mineral and bone disorders compromise bone formation and remodeling, also impacting growth. In addition, low birth weight and prematurity are common situations in these children that contribute to growth impairment^4^. Growth retardation related to CKD is, therefore, multifactorial. However, malnutrition is the most critical factor contributing to growth impairment, especially in young children^5^.

Knowing the modifiable factors and establishing strategies to improve care for these children is paramount. Growth failure progresses in parallel with CKD progression. However, the prevalence of short stature varies widely among countries, and most studies were conducted in economically developed countries. This research aims to analyze the prevalence of short stature in a sample of children with CKD not on dialysis in northeastern Brazil, and assess the risk factors associated with growth impairment in this population.

## Methods

This was a retrospective cohort study that evaluated pediatric outpatients with CKD aged between 1 and 18 years at the Renal Pediatric Unit of the Professor Fernando Figueira Institute of Integral Medicine (IMIP), Recife, Brazil. We reviewed the medical records of all patients (n = 49) undergoing regular follow-up between January and December 2020. The legal guardians of all children signed the informed consent. The local ethics committee approved the study by the number (CAAE #38877120.7.0000.5201). We included patients with stages 3, 4, or 5 CKD, defined as creatinine clearance < 60 mL/min /1.73m^2^, followed for at least one year. We excluded patients on dialysis.

Demographic data included age (in years) at baseline and follow-up, length of follow-up (in months), etiology of CKD, birth weight categorized as adequate (≥ 2.500g) or low weight (< 2.500g), gestational age categorized as a term (≥ 37 complete weeks) or preterm (≤ 36 weeks and six days). The anthropometric data analyzed included weight, height/length/age, weight/age, and body mass index (BMI), according to the growth charts of the World Health Organization (2006).

The laboratory tests evaluated were blood count, blood gas analysis, and biochemical markers of mineral and bone disease, including calcium, phosphate, alkaline phosphatase, intact parathormone (PTHi), and 25-hydroxy vitamin D. Normal values were considered according to recommendations K/DOQI guidelines^6^. The Schwartz formula was applied to calculate the glomerular filtration rate (GFR)^7^.

Continuous and semi-continuous data were analyzed using the Shapiro-Wilk test to determine normality. Mean and standard deviation or median and percentiles (25–75) were expressed as appropriate. The comparisons were performed by parametric and non-parametric tests according to data distribution. Categorical data were analyzed using the Chi-square with a significance test. We used the SPSS program (Statistical Package for the Social Sciences, version 25) for statistical analysis. All studies considered α ≤ 0.05 risk for type I error.

## Results

Out of the initial 49 patients, six were excluded due to missing data in the medical records. Therefore, we included 43 patients in the final analysis. The mean age at the end of the study period was 7.9 ± 4.6 years, the majority were boys (n = 28; 65%), and the median follow-up time was 36 (21–72) months. Etiology of kidney disease was congenital anomaly of the genitourinary tract in 38 (88.3%) patients (13 with posterior urethra valve, renal dysplasia in 8, neurogenic bladder in 6, polycystic kidney disease in 4, reflux nephropathy in 2, Prune-Belly syndrome in 2, anorectal anomaly in 1, and bladder exstrophy in 2, glomerulonephritis in 2 (4.7%), tubulopathy in 2 (4.7%) (1 with renal tubular acidosis and 1 with Type 1 Dent disease), and other causes (lymphangioma) in 1 (2.3%) patient. Table 1 summarizes demographic data.

**Table 1:** Clinical and demographic data of patients

Patients	n = 43
Male sex, (n, %)	28 (65)
Age, at the end of the study period (years)	7.9 ± 4.6
Birth weight (g)*(n, %)	3.018 ± 627.0
> 2500 g	29 (85)
≤ 2500 g	5 (15)
Gestational age**(n, %)	
≥ 37 weeks	26 (72.2)
< 37 weeks	10 (27.7)
Follow-up (months)	36 (21–72)
Etiology (n, %)	
Congenital anomaly	38 (88.3)
Glomerulopathy	2 (4.7)
Tubulopathy	2 (4.7)
Other	1 (2.3)

Note – Data are expressed as mean and SD, n (%) or median (25–75).

* n = 34,

** n = 36.

**Figure 1:**
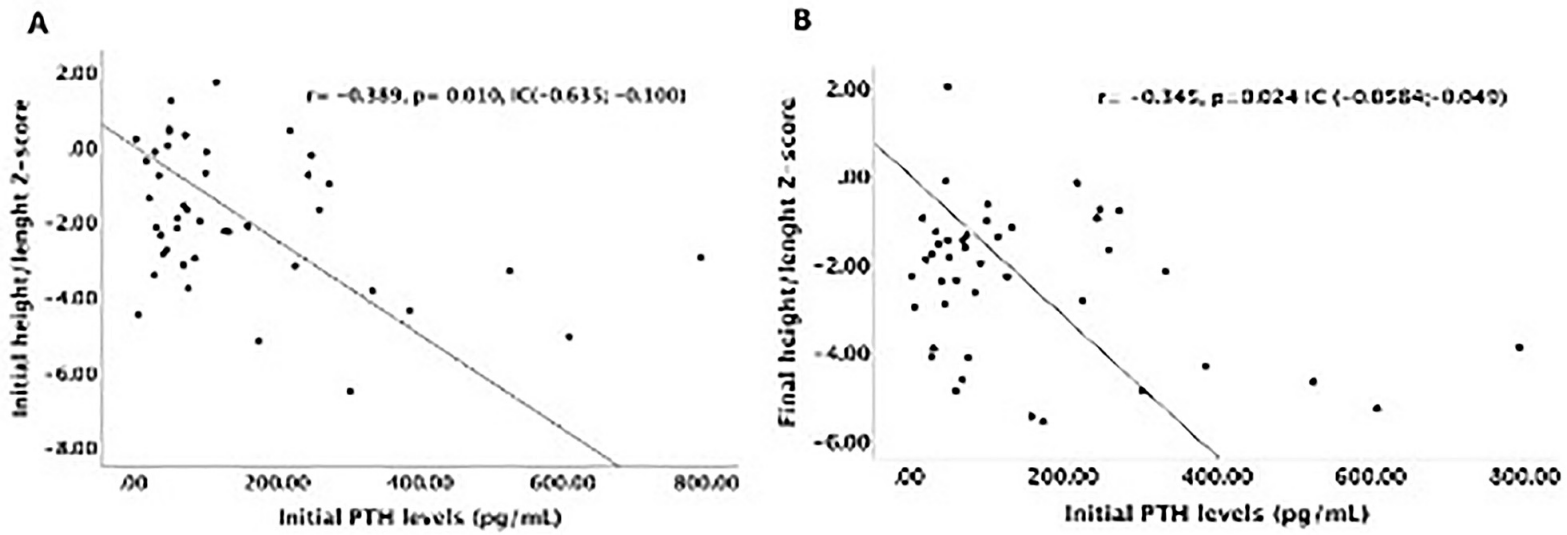
Correlation between initial PTH levels and height/length z-score.

At baseline and the end of follow-up, there were 22 (51%) and 21 (49%) children with short stature (p = 0.82), respectively. During follow-up, the height/length/age increased, which was not verified by the height/length/age z-score (Table 2). BMI increased during follow-up, although this was not confirmed by BMI/age z-score [from –0.62 (–1.4 – 0.33) to –0.25 (–0.82 – 0.84), p = 0.21]. Laboratory data revealed a worsening serum creatinine and PTH and improved bicarbonate and hemoglobin levels during the study period. When we evaluated serum levels according to age group during follow-up, alkaline phosphatase was elevated in 63% of patients, 10% had hyperphosphatemia, and 5% had hypophosphatemia (Table 2). None of the 43 patients underwent recombinant growth hormone (GH) therapy. Table 3 shows that children with short stature were younger and had lower BMI/age than those with adequate stature. However, there were no differences between the groups when we analyzed the BMI/age z-score. Moreover, more children with short stature had serum phosphate outside the normal range (p = 0.035). Analysis of the glomerular filtration rate, markers of bone mineral disease, bicarbonate, and hemoglobin showed that serum PTH levels at the beginning of follow-up correlated with the baseline height/length/age z-score (r = –0.389; p = 0.010) and final height/length/age z-score (r = –0.345; p = 0.024) (Figure 1). For the other laboratory data analyzed, this correlation was not verified.

**Table 2:** Initial and final anthropometric and laboratory data of patients

Parameter	Initial	Final	P
Height/length/age (cm)	90.6 ± 3.9	112.4 ± 30.8	0.000
Height/length/age z-score	–1.8 ± 1.8	–2.4 ± 1.6	0.011
Short stature (n, %)	22 (51)	21 (49)	0.820
BMI/age	14.2 (13.0 – 17.0)	15.7 (14.8 – 18.2)	0.040
BMI/age z-score	–0.6 (–1.4 – 0.33)	–0.2 (–0.82 – 0.84)	0.210
Adequate (n, %)	30 (70)	30 (70)	1.000
Underweight	6 (14)	6 (14)	
Overweight/Obesity	7 (16)	7 (16)	
Urea, (mg/dL)	78.5 ± 37.9	85.4 ± 34.9	0.338
Creatinine (mg/dL)	1.4 (0.9 – 1.9)	1.8 (1.4 – 2.8)	0.003
Calcium (mg/dL)	9.8 (9.3 – 10.5)	9.7 (9.3 – 10.1)	0.194
Normal for age (n, %)	28 (65)	33 (77)	0.814
Elevated for age	12 (28)	8 (19)	
Low for age	3 (7)	2 (4)	
Phosphate (mg/dL)	5.3 (4.7 – 5.9)	5.0 (4.5 – 5.7)	0.109
Normal for age (n, %)	32 (75)	30 (70)	0.014
Elevated for age	11 (25)	10 (23)	
Low for age	0 (0)	3 (7)	
Alkaline Phosphatase (U/L)	302 (210 – 473)	317 (207 – 495)	0.587
Normal for age (n, %)	30 (70)	16 (37)	0.001
Elevated for age	13 (30)	27 (63)	
Parathormone (pg/mL)	76 (45.6 – 225.5)	136.7 (90.3 – 218.5)	0.034
Bicarbonate (mEq/L)	18.2 ± 4.5	20.7 ± 4.0	0.004
< 22 mEq/L (n, %)	32 (74.4)	26 (60.5)	0.160
Hemoglobin (g/dL)	10.2 ± 1.7	11.5 ± 1.9	0.000
Anemia (n, %)	21 (48.8)	8 (18.6)	0.001

Note – Data are expressed as n (%), mean and SD or median, 25–75% range. Abbreviations – BMI: body mass index; CKD: chronic kidney disease.

**Table 3:** Anthropometric and laboratory data among children with short and adequate stature at the end of the study period

	Adequate staturen = 22	Short staturen = 21	P
Age at the end of the study period (years)	10.5 (5.7 – 12.0)	5 (2.0 – 10.0)	0.027
Gestational age*			
≥ 37 weeks	14 (38.8)	12 (33.3)	0.199
< 37 weeks	3 (8.3)	7 (19.4)	
Birth weight**			
> 2500g	14 (41.1)	15 (44.1)	0.240
≤ 2500g	1 (2.9)	4 (11.7)	
Sex			
Boy	17 (39.5)	11 (25.5)	0.087
Girl	5 (11.6)	10 (23.2)	
Follow-up (months)	47 ± 30.6	42 ± 28.7	0.582
Height/length/for age (cm)	125.0 ± 27.6	98.3 ± 28.0	0.000
Height/length/age z-score	–1.1 (–1.6 – –0.8)	–3.9 (–4.7 – –2.5)	0.001
BMI/age	16.7 (15.5 – 19.6)	15.1 (4.3 – 16.9)	0.023
BMI/age z-score	0.1 ± 1.4	–0.6 ± 1.4	0.094
Adequate (n, %)	14 (32.5)	16 (37.2)	0.114
Underweight	2 (4.6)	4 (9,3)	
Overweight/Obesity	6 (13.9)	1 (2.3)	
Urea (mg/dL)	78.7 ± 29.1	92.4 ± 39.6	0.204
Creatinine (mg/dL)	1.4 (1.0 – 1.9)	1.4 (0.9 – 1 .8)	0.307
CKD stage			
III	14	10	0.425
IV	7	8	
V	1	3	
GFR (mL/min/1.73m^2^)	34.6 ± 15.1	30.9 ± 13.5	0.411
Calcium (mg/dL)	9.8 (9.5 – 10.2)	9.4 (8.8 – 10.0)	0.059
Phosphate (mg/dL)	5.0 ± 0.5	5.0 ± 1.5	0.862
Normal for age (n, %)	19 (44.1)	11 (25.5)	0.035
Elevated for age	3 (6.9)	7 (16.2)	
Low for age	0 (0.0)	3 (6.9)	
Alkaline phosphatase (U/L)	319 (230 – 399)	274 (207 – 563)	0.971
Normal for age (n, %)	9 (20.9)	7 (16.2)	0.607
Elevated for age	13 (30.2)	14 (32.5)	
Parathormone (pg/mL)	138 (95 – 227)	132 (64 – 303)	0.789
Bicarbonate (mEq/L)	21 (20 – 23)	20 (19 – 22)	0.103
< 22mEq/L (n,%)	11(50.0)	15 (71.4)	0.151
Hemoglobin (g/dL)	11.0 ± 1.6	11.1 ± 2.2	0.239
Anemia (n, %)	2 (9.0)	6 (28.5)	0.101
Albumin (g/dL)	4.5 (4.3 – 4.6)	4.5 (4.1 – 4.8)	0.864
25OH vitamin D (ng/mL)	41 (34.2 – 44.2)	40 (30.5 – 47.5)	0.942

Note – Data are expressed as (n, %), mean and SD, or median, 25–75% range,

* n = 36,

** n = 34.

Abbreviations – GFR: glomerular filtration rate.

A sub-analysis with children under five years old at the end of the study period (n = 17) showed that 10 (58.8%) had impaired growth. Although these children had lower creatinine (p = 0.032) and lower urea (p = 0.015), they showed lower weight/age z-score (0.031) and lower BMI/age z-score (p = 0.04). A difference in serum phosphate was not observed when analyzed by age group (Table 4).

**Table 4:** Anthropometric and laboratory data of children age five years or younger whose growth has improved and whose growth was maintained or declined

	Improved growthn = 7	Maintained or declined Growthn = 10	P
Age at the end of the study period (years)	5 (2.0 – 5.0)	2 (1.7 – 2.5)	0.08
Sex (n, %)			
Boy	5 (29.4)	6 (35.2)	0.62
Girl	2 (11.7)	4 (23.5)	
Follow–up (months)	29 (21.0 – 60.0)	21 (9.0 – 33.7)	0.18
Height/length/age (cm)	89.3 ± 10.5	79.9 ± 15.6	0.18
Height/length/age z–score	–2.9 ± 1.3	–3.2 ± 1.9	0.69
BMI/for age	16.9 ± 1.6	15.2 ± 1.8	0.71
BMI/age z–score	0.95 ± 1.1	–0.47 ± 1.6	0.04
Adequate (n, %)	5 (29.4)	7 (41.1)	0.33
Underweight	0 (0.0)	2 (11.7)	
Overweight/Obesity	2 (11.7)	1(5.8)	
Urea (mg/dL)	115.0 ± 37.0	66.0 ± 35.6	0.01
Creatinine (mg/dL)	2.3 (1.4 – 3.2)	1.1(0.8 – 1.6)	0.03
CKD stage			
III	2	6	0.25
IV	2	3	
V	3	1	
Glomerular filtration rate (mL/min/1.73m^2^)	22.5 ± 14.1	38.0 ± 19.0	0.74
Bicarbonate (mEq/L)	20 (11 – 25)	21 (18 – 22)	0.08
< 22 mEq/L (n, %)	2 (11.7)	3 (17.6)	0.94
Hemoglobin (g/dL)	11.6 ± 2.0	10.5 ± 2.2	0.33
Anemia (n, %)	2 (11.7)	2 (11.7)	0.68
Calcium (mg/dL)	9.4 ± 1.6	9.7 ± 0.8	0.67
Phosphate (mg/dL)	5.7 (5.7 – 6.7)	4.7 (4.3 – 5.7)	0.02
Normal for age (n, %)	5 (29.4)	7 (41.1)	0.66
Elevated for age	2 (11.7)	2 (11.7)	
Low for age	0 (0.0)	1(5.9)	
Alkaline phosphatase (U/L)	343 ± 207	362 ± 200	0.84
Normal for age (n, %)	2 (11.7)	5 (29.4)	0.37
Elevated for age	5 (29.4)	5 (29.4)	
Parathormone (pg/mL)	120 (51 – 361)	125 (77 – 414)	0.92
Albumin (g/dL)	4.2 ± 0.5	4.2 ± 0.7	0.95
25OH vitamin D (ng/mL)	40.8 ± 14.7	43.5 ± 6.6	0.62

Note – Data are expressed as (n, %), mean and SD, or median, range 25–75%.

## Discussion

Growth failure is one of the main complications of CKD in children. Each unit decrease in height/length-for-age z-score is associated with a 14% increase in mortality of affected children^8^. In general, the prevalence of short stature in children with CKD varies from 30 to 50%^9^. The PICCOLO MONDO study of 225 children from cities in four continents, including the Brazilian city of Curitiba, found that 39% of Latin American children on hemodialysis had short stature^9^. In our series, we found that 51% of children had short stature, which was highly prevalent in younger children, including the subset of children under five. These data contrast with the results of Brazilian populational studies such as the National Demographic and Health Survey (PNDS) and the III State Health and Nutrition Survey (PESN), which show a prevalence of short stature in the general population of children under five years of age of 7.0% in Brazil, 5.7% in the Northeast region and 8.7% in the state of Pernambuco^10^.

Malnutrition might play a role in these scenarios. Nonetheless, children with CKD present an additional risk of malnutrition as they can maintain high resting energy expenditure while losing lean body mass and maintaining body fat mass, leading to protein-energy wasting (PEW) and resulting in growth impairment. Catch-up growth may be incomplete even in those children receiving optimal calorie and protein intake^11^. Even so, optimizing caloric intake in children with CKD is the most effective strategy to improve growth^1^. Ensuring adequate and appropriate nutritional intake is essential, particularly in young children. A recent study showed a benefit in weight and height/length gain in children fed by enteral tube and considered its use for children with CKD under six years old with low BMI^12^.

Furthermore, conditions specific to CKD, such as mineral and bone disorders, contribute to short stature in these children. Phosphate is known as a “silent killer” because of its effect on mineral and bone disorders and its association with vascular calcifications. However, phosphate intake is associated with protein intake^13^. Our data demonstrated that children with short stature had worse phosphate control than their counterparts. Despite our clinical routine of dietary guidelines and phosphorus-chelating medications, patients had hyperphosphatemia, probably due to protein intake and phosphate overload “hidden” in food additives. The nutritional approach to CKD aims to provide enough protein to avoid malnutrition and allow proper growth, while avoiding hyper and hypophosphatemia. Our results highlight that the nutrition of these patients, should be better monitored, ensuring the supply of proteins and controlling phosphate intake.

Another risk factor for growth retardation is secondary hyperparathyroidism in CKD, as it may destroy the metaphyseal bone architecture and result in growth arrest^14^. CKD causes bone resistance to PTH, increasing its levels in the early stages of the disease^15^. Some authors argue that this increase is an appropriate adaptive response to the decline in renal function. They show a positive association between PTH and growth potential^16^. Other researchers suggest that higher PTH levels (up to twice the expected value for age) are associated with better growth in these children^17^. We observed elevated serum PTH levels during the study follow-up. As for alkaline phosphatase, 63% of children showed high serum levels for their age during the study period. Moreover, we observed an inverse correlation between initial PTH levels and the height/age z-score assessed at the beginning and end of the follow-up period. These results corroborate the diagnosis of CKD mineral and bone disorder and their negative impact on the growth of children with CKD.

Metabolic acidosis is known to degrade proteins, increase endogenous production of corticosteroids, reduce growth cartilage, reduce the secretion of growth hormone (GH) and increase resistance to it^18^. Furthermore, acidosis is associated with higher mortality in patients with CKD, demonstrating the importance of adequate control. The 4C study (Cardiovascular Comorbidity in Children with CKD) in 704 children with CKD showed a prevalence of metabolic acidosis in 43%, 60%, and 45% in CKD 3, 4, and 5, respectively, and bicarbonate levels below 18 mEq/L were not associated with growth^19^. Our analysis did not show any association between bicarbonate levels and growth. Despite these results, metabolic acidosis was present in 74% of patients at baseline and in 60% at the end of the follow-up. In our practice, we prescribe sodium bicarbonate. However, many of our patients have difficulty accessing medications and lack treatment adherence. In addition, there are reports that medical commitment impacts the growth of children with CKD^20^. These data reinforce the need to develop strategies to improve the adherence of these patients and strict surveillance to maintain adequate levels of this ion.

Another factor that impacts the growth of children with CKD is anemia. Forty-nine percent of our patients had anemia at the beginning of the follow-up. We observed an improvement in hemoglobin levels at the end of the study period, probably due to treatment. However, anemia persisted in 18% of cases. The North American Pediatric Renal Transplant Cooperative Studies (NAPRTCS) report showed that 46% of children had anemia at the beginning of the study, which was associated with growth retardation. Boehm et al.^21^ observed an improvement in growth in only 40% of children under erythropoietin treatment. This fact draws our attention to the point that we still need to improve the surveillance and adequacy of the treatment of anemia. At the same time, it may also reflect the evolution of CKD itself.

The association between low birth weight, prematurity, and short stature in children with CKD is already known^22^. Greenbaum et al.^23^ showed low birth weight in 17% of children with CKD, which was similar to our finding (n = 5, 14.7%). However, we did not find an association with stature.

The stage of CKD can impact growth, and children in the early stages of the disease are more likely to have adequate stature. Factors implicated in the pathogenesis and CKD progression may be involved in growth failure in these cases^24^. Regardless addition to renal function, the time of illness may effect growth. Ingulli and Mak^25^ observed that children with CKD had progressive growth retardation. Similar to that study, our results showed a worsening in children’s height z-score (–1.89 ± 1.84 and –2.4 ± 1.67, p = 0.011) during the 36-month follow-up.

Here, we call attention to the characteristics of the studied population. It is part of our daily practice (data not shown) to assist children from low-income families who arrive at the service in an advanced stage of the disease, come from cities far from the center, and, therefore have difficulty in maintaining regular follow-up. It is crucial to highlight the impact of socioeconomic conditions on CKD outcomes^26^. Low socioeconomic level of the families implies in lack of knowledge of the disease, low adherence, and lack of resources to purchase medications, and it is a common problem in our country^27^.

The positive effect of using GH on the final height of children with CKD is already well documented^28^. The most recent European guidelines recommend GH therapy in short-stature children with CKD stage 3–5D, suggesting the modification of risk factors before its use^29^. Unfortunately, despite the benefits of GH, it is still underused in children with CKD. A similar situation was present in our population, where no patient underwent recombinant GH therapy. This worrying scenario shows the importance of implementing a more effective access policy for this therapy.

Early interventions are essential and much more effective if they start before dialysis. Haffner^30^ recently published strategies to guide the prevention and treatment of growth retardation in these children with CKD. The guidelines suggest focusing on preserving renal function through the normalization of blood pressure and proteinuria with the use of inhibitors of the renin-angiotensin-aldosterone system, treating anemia with erythropoiesis stimulators, taking care of energy adequacy, including the use of an enteral tube or gastrostomy when necessary, correcting hydro-electrolytic and acid-base disturbances, controlling PTH levels in the target range of CKD, using recombinant GH when indicated, and early kidney transplantation with immunosuppression protocols with minimal doses of steroids^30^.

The limitations of our study were its retrospective, single-center nature and small sample size, which limited us from expanding the analyses. However, it revealed a high prevalence of short stature, which persisted during the follow-up, especially in young children. Moreover, PTH levels were inversely correlated with the height/age z-score, and growth failure in these children was associated with worse nutritional status. Our results reinforce the importance of monitoring the growth of these children, maintaining control of hyperparathyroidism, and providing nutritional support.

## References

[1] Silverstein DM (2018). Growth and nutrition in pediatric chronic kidney disease. Front Pediatr.

[2] Fernández-Iglesias Á, López JM, Santos F (2020). Growth plate alterations in chronic kidney disease. Pediatr Nephrol.

[3] Atkinson MA, Warady BA (2018). Anemia in chronic kidney disease. Pediatr Nephrol.

[4] Wesseling-Perry K (2015). Defective skeletal mineralization in pediatric CKD. Curr Osteoporos Rep.

[5] Rees L, Mak RH (2011). Nutrition and growth in children with chronic kidney disease. Nat Rev Nephrol.

[6] National Kidney Foundation (2003). Am J Kidney Dis.

[7] Schwartz GJ, Muñoz A, Schneider MF, Mak RH, Kaskel F, Warady BA (2009). New equations to estimate GFR in children with CKD. J Am Soc Nephrol.

[8] Furth SL, Hwang W, Yang C, Neu AM, Fivush BA, Powe NR (2002). Growth failure, risk of hospitalization and death for children with end-stage renal disease. Pediatr Nephrol.

[9] Hussein R, Alvarez-Elías AC, Topping A, Raimann JG, Filler G, Yousif D, MONDO Consortium (2018). A cross-sectional study of growth and metabolic bone disease in a pediatric global cohort undergoing chronic hemodialysis. J Pediatr.

[10] Leal VS, Lira PIC, Menezes RCE, Oliveira JS, Sequeira LAS, Andrade SLLS (2012). Fatores associados ao declínio do déficit estatural em crianças e adolescentes em Pernambuco. Rev Saude Publica.

[11] Rees L (2021). Protein energy wasting; what is it and what can we do to prevent it?. Pediatr Nephrol.

[12] Marlais M, Stojanovic J, Jones H, Cleghorn S, Rees L (2020). Catch-up growth in children with chronic kidney disease started on enteral feeding after 2 years of age. Pediatr Nephrol.

[13] Bacchetta J, Bernardor J, Garnier C, Naud C, Ranchin B (2021). Hyperphosphatemia and chronic kidney disease: a major daily concern both in adults and in children. Calcif Tissue Int.

[14] Santos F, Díaz-Anadón L, Ordóñez FA, Haffner D (2021). Bone disease in CKD in children. Calcif Tissue Int.

[15] Wesseling-Perry K, Pereira RC, Tseng CH, Elashoff R, Zaritsky JJ, Yadin O (2012). Early skeletal and biochemical alterations in pediatric chronic kidney disease. Clin J Am Soc Nephrol.

[16] Haffner D, Leifheit-Nestler M (2020). Treatment of hyperphosphatemia: the dangers of aiming for normal PTH levels. Pediatr Nephrol.

[17] Bacchetta J (2020). Treatment of hyperphosphatemia: the dangers of high PTH levels. Pediatr Nephrol.

[18] Kraut JA, Madias NE (2011). Consequences and therapy of the metabolic acidosis of chronic kidney disease. Pediatr Nephrol.

[19] Harambat J, Kunzmann K, Azukaitis K, Bayazit AK, Canpolat N, Doyon A, 4C Study Consortium (2017). Metabolic acidosis is common and associates with disease progression in children with chronic kidney disease. Kidney Int.

[20] Akchurin OM, Schneider MF, Mulqueen L, Brooks ER, Langman CB, Greenbaum LA (2014). Medication adherence and growth in children with CKD. Clin J Am Soc Nephrol.

[21] Boehm M, Riesenhuber A, Winkelmayer WC, Arbeiter K, Mueller T, Aufricht C (2007). Early erythropoietin therapy is associated with improved growth in children with chronic kidney disease. Pediatr Nephrol.

[22] Franke D, Alakan H, Pavicˇic´ L, Gellermann J, Müller D, Querfeld U (2013). Birth parameters and parental height predict growth outcome in children with chronic kidney disease. Pediatr Nephrol.

[23] Greenbaum LA, Muñoz A, Schneider MF, Kaskel FJ, Askenazi DJ, Jenkins R (2011). The association between abnormal birth history and growth in children with CKD. Clin J Am Soc Nephrol.

[24] Behnisch R, Kirchner M, Anarat A, Bacchetta J, Shroff R, Bilginer Y, 4C Study Consortium (2019). Determinants of statural growth in european children with chronic kidney disease: findings from the cardiovascular comorbidity in children with chronic kidney disease (4C) study. Front Pediatr.

[25] Ingulli EG, Mak RH (2014). Growth in children with chronic kidney disease: role of nutrition, growth hormone, dialysis, and steroids. Curr Opin Pediatr.

[26] Atkinson MA, Ng DK, Warady BA, Furth SL, Flynn JT (2021). The CKiD study: overview and summary of findings related to kidney disease progression. Pediatr Nephrol.

[27] de Pádua Paz I, Konstantyner T, de Castro Cintra Sesso R, de Xavier Pinto CC, de Camargo MFC, Nogueira PCK (2021). Access to treatment for chronic kidney disease by children and adolescents in Brazil. Pediatr Nephrol.

[28] Drube J, Wan M, Bonthuis M, Wühl E, Bacchetta J, Santos F (2019). Clinical practice recommendations for growth hormone treatment in children with chronic kidney disease. Nat Rev Nephrol.

[29] Hodson EM, Willis NS, Craig JC (2012). Growth hormone for children with chronic kidney disease. Cochrane Database Syst Rev.

[30] Haffner D (2020). Strategies for optimizing growth in children with chronic kidney disease. Front Pediatr.

