# Supplementary material for: Growth in children with chronic kidney disease and associated risk factors for short stature
Source: J Bras Nefrol. 2024 Jul 29;46(4):e20230203. doi: 10.1590/2175-8239-JBN-2023-0203en (PMC11305564; doi:10.1590/2175-8239-JBN-2023-0203en)
Supplement: Translation

# Translation: Crescimento em crianças com doença renal crônica e fatores de risco associados à baixa estatura

**DOI:** 10.1590/2175-8239-JBN-2023-0203pt

**Authors:** Virgínia Barbosa de Melo, Danielle Barbosa da Silva, Matheus Dantas Soeiro, Lucas Cavalcante Tenório de Albuquerque, Henderson Edward Firmino Cavalcanti, Marcela Correa Araújo Pandolfi, Rosilene Mota Elias, Rosa Maria Affonso Moysés, Emília Maria Dantas Soeiro

**Affiliations:** 1 Instituto de Medicina Integral Professor Fernando Figueira, Recife, PE, Brasil.; 2 Faculdade Pernambucana de Saúde, Recife, PE, Brasil.; 3 Universidade de São Paulo, Hospital das Clínicas, São Paulo, SP, Brasil.; 4 Universidade de São Paulo, Faculdade de Medicina, Laboratório de Investigação Médica, São Paulo, SP, Brasil.; 5 Universidade Federal de Pernambuco, Faculdade de Medicina do Recife, Recife, PE, Brasil.

**Keywords:** Insuficiência Renal Crônica, Insuficiência de Crescimento, Distúrbio Mineral e Ósseo na Doença Renal Crônica, Crescimento, Nutrição da Criança.

## Abstract

**Introdução:**

A deficiência de crescimento na doença renal crônica está relacionada à elevada morbidade e mortalidade. O retardo do crescimento nessa doença é multifatorial. É fundamental conhecer os fatores modificáveis e estabelecer estratégias para melhorar o atendimento às crianças afetadas.

**Objetivos:**

Descrever padrões de crescimento em crianças com DRC e fatores de risco associados à baixa estatura.

**Métodos:**

Analisamos retrospectivamente dados antropométricos e epidemiológicos, peso ao nascer, prematuridade e níveis de bicarbonato, hemoglobina, cálcio, fosfato, fosfatase alcalina e paratormônio de crianças com DRC estágios 3–5, não submetidas a diálise, acompanhadas por pelo menos um ano.

**Resultados:**

Incluímos 43 crianças, a maioria meninos (65%). O escore z médio de altura/comprimento/idade das crianças no início e no acompanhamento foi de –1,89 ± 1,84 e –2,4 ± 1,67, respectivamente (p = 0,011). Cinquenta e um por cento das crianças apresentaram baixa estatura, e essas crianças eram mais jovens que aquelas com estatura adequada (p = 0,027). Níveis de PTH no início do acompanhamento correlacionados com escore z de altura/comprimento/idade. Uma subanálise com crianças menores de cinco anos (n = 17) mostrou que 10 (58,8%) delas apresentaram déficit de desenvolvimento e escore z de peso/idade mais baixo (0,031) e escore z de IMC/idade mais baixo (p = 0,047).

**Conclusão:**

Crianças, especialmente mais jovens, com DRC, que não estavam em diálise, apresentaram elevada prevalência de baixa estatura. Níveis de PTH foram correlacionados com escore z de altura, e deficiência de crescimento foi associada a pior estado nutricional. Portanto, é essencial monitorar o crescimento dessas crianças, controlar o hiperparatireoidismo e fornecer suporte nutricional.

## Introdução

O retardo do crescimento no contexto da doença renal crônica (DRC) causa múltiplos desfechos desfavoráveis, incluindo baixa qualidade de vida, baixa autoestima, pior desempenho escolar e alta morbidade e mortalidade^1^. As alterações nas proteínas de ligação ao fator de crescimento semelhante à insulina (IGFBPs, por sua sigla em inglês) em crianças com DRC levam a uma atividade reduzida de IGF nos condrócitos da placa de crescimento. Esse mecanismo ocorre devido à competição com os receptores de IGF tipo 1 e ajuda a explicar a resistência ao hormônio do crescimento (GH, na sigla em inglês)^2^.

Os fatores associados à DRC, como deficiência nutricional, acidose metabólica, anemia e doença mineral óssea, agravam a condição dessas crianças. As restrições alimentares impostas pelo tratamento da DRC e a ingestão reduzida secundária à doença contribuem para a deficiência nutricional^1^. A acidose metabólica é um estímulo negativo na secreção do GH^1^. A anemia prejudica a oxigenação celular e afeta o crescimento^3^. Distúrbios minerais e ósseos comprometem a formação e a remodelação óssea, afetando também o crescimento. Além disso, o baixo peso ao nascer e a prematuridade são situações comuns nessas crianças que contribuem para o comprometimento do crescimento^4^. O retardo do crescimento relacionado à DRC é, portanto, multifatorial. No entanto, a desnutrição é o fator mais importante que contribui para o comprometimento do crescimento, especialmente em crianças pequenas^5^.

É fundamental conhecer os fatores modificáveis e estabelecer estratégias para melhorar o atendimento a essas crianças. O déficit de crescimento progride paralelamente à progressão da DRC. No entanto, a prevalência de baixa estatura varia consideravelmente entre os países, e a maioria dos estudos foi realizada em países economicamente desenvolvidos. Esta pesquisa tem como objetivo analisar a prevalência de baixa estatura em uma amostra de crianças com DRC não submetidas à diálise no Nordeste do Brasil e avaliar os fatores de risco associados ao comprometimento do crescimento nessa população.

## Métodos

Este foi um estudo de coorte retrospectivo que avaliou pacientes ambulatoriais pediátricos com DRC com idades entre 1 e 18 anos na Unidade Renal Pediátrica do Instituto de Medicina Integral Professor Fernando Figueira (IMIP), Recife, Brasil. Revisamos os prontuários médicos de todos os pacientes (n = 49) submetidos a acompanhamento regular entre janeiro e dezembro de 2020. Os responsáveis legais de todas as crianças assinaram o consentimento informado. O comitê de ética local aprovou o estudo pelo número (CAAE #38877120.7.0000.5201). Foram incluídos pacientes com DRC estágios 3, 4 ou 5, definidos como *clearance* de creatinina < 60 mL/min /1,73m^2^, acompanhados por pelo menos um ano. Foram excluídos os pacientes em diálise.

Os dados demográficos incluíram idade (em anos) no início e no acompanhamento, duração do acompanhamento (em meses), etiologia da DRC, peso ao nascer categorizado como adequado (≥ 2.500g) ou baixo peso (< 2.500g), idade gestacional categorizada como a termo (≥ 37 semanas completas) ou pré-termo (≤ 36 semanas e seis dias). Os dados antropométricos analisados incluíram peso, altura/comprimento/idade, peso/idade e índice de massa corporal (IMC), de acordo com os gráficos de crescimento da Organização Mundial da Saúde (2006).

Os exames laboratoriais avaliados foram hemograma, gasometria e marcadores bioquímicos de doença mineral e óssea, incluindo cálcio, fosfato, fosfatase alcalina, paratormônio intacto (PTHi) e 25-hidroxi vitamina D. Os valores normais foram considerados de acordo com as recomendações das diretrizes do K/DOQI^6^. A fórmula de Schwartz foi aplicada para calcular a taxa de filtração glomerular (TFG)^7^.

Os dados contínuos e semicontínuos foram analisados por meio do teste de Shapiro-Wilk para determinar a normalidade. Média e desvio padrão ou mediana e percentis (25–75) foram expressos conforme apropriado. As comparações foram realizadas por testes paramétricos e não paramétricos de acordo com a distribuição dos dados. Os dados categóricos foram analisados utilizando o teste qui-quadrado com um teste de significância. Utilizamos o programa SPSS (*Statistical Package for the Social Sciences*, versão 25) para análise estatística. Todos os estudos consideraram um risco α ≤ 0,05 para erro tipo I.

## Resultados

Dos 49 pacientes iniciais, seis foram excluídos devido à falta de dados nos registros médicos. Portanto, foram incluídos 43 pacientes na análise final. A média de idade ao final do período do estudo foi de 7,9 ± 4,6 anos, a maioria era de meninos (n = 28; 65%) e o tempo mediano de acompanhamento foi de 36 (21–72) meses. A etiologia da doença renal foi anomalia congênita do trato geniturinário em 38 (88,3%) pacientes (13 com válvula de uretra posterior, displasia renal em 8, bexiga neurogênica em 6, doença renal policística em 4, nefropatia de refluxo em 2, síndrome de Prune-Belly em 2, anomalia anorretal em 1 e extrofia de bexiga em 2), glomerulonefrite em 2 (4,7%), tubulopatia em 2 (4,7%) (1 com acidose tubular renal e 1 com doença de Dent tipo 1) e outras causas (linfangioma) em 1 (2,3%) paciente. A Tabela 1 resume os dados demográficos.

**Tabela 1:** Dados clínicos e demográficos dos pacientes

Pacientes	n = 43
Sexo masculino, (n, %)	28 (65)
Idade, ao final do período do estudo (anos)	7,9 ± 4,6
Peso ao nascer (g)*(n, %)	3,018 ± 627,0
> 2500 g	29 (85)
≤ 2500 g	5 (15)
Idade gestacional**(n, %)	26 (72,2)
≥ 37 semanas	10 (27,7)
< 37 semanas	
Acompanhamento (meses)	36 (21–72)
Etiologia (n, %)	38 (88,3)
Anomalia congênita	2 (4,7)
Glomerulopatia	2 (4,7)
Tubulopatia	1 (2,3)
Outras	

Nota – Os dados são expressos como média e DP, n (%) ou mediana (25–75).

* n = 34,

** n = 36.

No início e no final do acompanhamento, havia 22 (51%) e 21 (49%) crianças com baixa estatura (p = 0,82), respectivamente. Durante o acompanhamento, houve aumento da altura/comprimento/idade, o que não foi verificado pelo escore z de altura/comprimento/idade (Tabela 2). O IMC aumentou durante o acompanhamento, embora isso não tenha sido confirmado pelo escore z de IMC/idade [de –0,62 (–1,4 – 0,33) para –0,25 (–0,82 – 0,84), p = 0,21]. Os dados laboratoriais revelaram uma piora nos níveis de creatinina sérica e PTH e uma melhora nos níveis de bicarbonato e hemoglobina durante o período do estudo. Quando avaliamos os níveis séricos de acordo com a faixa etária durante o acompanhamento, a fosfatase alcalina estava elevada em 63% dos pacientes, 10% apresentaram hiperfosfatemia e 5% apresentaram hipofosfatemia (Tabela 2). Nenhum dos 43 pacientes foi submetido à terapia com hormônio do crescimento (GH) recombinante. A Tabela 3 mostra que crianças com baixa estatura eram mais jovens e apresentavam menor IMC/idade do que as com estatura adequada. No entanto, não houve diferenças entre os grupos quando analisamos o escore z de IMC/idade. Além disso, mais crianças com baixa estatura apresentaram fosfato sérico fora da faixa normal (p = 0,035). A análise da taxa de filtração glomerular, marcadores de doença mineral óssea, bicarbonato e hemoglobina mostraram que os níveis séricos de PTH no início do acompanhamento se correlacionaram com o escore z basal de altura/comprimento/idade (r = –0,389; p = 0,010) e o escore z final de altura/comprimento/idade (r = –0,345; p = 0,024) (Figura 1). Para os demais dados laboratoriais analisados, essa correlação não foi verificada.

**Tabela 2:** Dados antropométricos e laboratoriais iniciais e finais dos pacientes

Parâmetro	Inicial	Final	P
Altura/comprimento/idade (cm)	90,6 ± 3,9	112,4 ± 30,8	0,000
Escore z de altura/comprimento/idade	–1,8 ± 1,8	–2,4 ± 1,6	0,011
Baixa estatura (n, %)	22 (51)	21 (49)	0,820
IMC/idade	14,2 (13,0 – 17,0)	15,7 (14,8 – 18,2)	0,040
Escore z de IMC/idade	–0,6 (–1,4 – 0,33)	–0,2 (–0,82 – 0,84)	0,210
Adequado (n, %)	30 (70)	30 (70)	1,000
Abaixo do peso	6 (14)	6 (14)	
Sobrepeso/obesidade	7 (16)	7 (16)	
Ureia, (mg/dL)	78,5 ± 37,9	85,4 ± 34,9	0,338
Creatinina (mg/dL)	1,4 (0,9 – 1,9)	1,8 (1,4 – 2,8)	0,003
Cálcio (mg/dL)	9,8 (9,3 – 10,5)	9,7 (9,3 – 10,1)	0,194
Normal para a idade (n, %)	28 (65)	33 (77)	0,814
Elevado para a idade	12 (28)	8 (19)	
Baixo para a idade	3 (7)	2 (4)	
Fosfato (mg/dL)	5,3 (4,7 – 5,9)	5,0 (4,5 – 5,7)	0,109
Normal para a idade (n, %)	32 (75)	30 (70)	0,014
Elevado para a idade	11(25)	10 (23)	
Baixo para a idade	0 (0)	3 (7)	
Fosfatase alcalina (U/L)	302 (210 – 473)	317 (207 – 495)	0,587
Normal para a idade (n, %)	30 (70)	16 (37)	0,001
Elevado para a idade	13 (30)	27 (63)	
Paratormônio (pg/mL)	76 (45,6 – 225,5)	136,7 (90,3 – 218,5)	0,034
Bicarbonato (mEq/L)	18,2 ± 4,5	20,7 ± 4,0	0,004
< 22 mEq/L (n, %)	32 (74,4)	26 (60,5)	0,160
Hemoglobina (g/dL)	10,2 ± 1,7	11,5 ± 1,9	0,000
Anemia (n, %)	21 (48,8)	8 (18,6)	0,001

Nota – Os dados são expressos como n (%), média e DP ou mediana, intervalo de 25–75%. Abreviações – IMC: índice de massa corporal; DRC: doença renal crônica.

**Tabela 3:** Dados antropométricos e laboratoriais entre crianças com baixa estatura e estatura adequada ao final do período do estudo

	Estatura adequada n = 22	Baixa estatura n = 21	P
Idade ao final do período do estudo (anos)	10,5 (5,7 – 12,0)	5 (2,0 – 10,0)	0,027
Idade gestacional*			
≥ 37 semanas	14 (38,8)	12 (33,3)	0,199
< 37 semanas	3 (8,3)	7 (19,4)	
Peso ao nascer**			
> 2500g	14 (41,1)	15 (44,1)	0,240
≤ 2500g	1 (2,9)	4 (11,7)	
Sexo			
Menino	17 (39,5)	11 (25,5)	0,087
Menina	5 (11,6)	10 (23,2)	
Acompanhamento (meses)	47 ± 30,6	42 ± 28,7	0,582
Altura/comprimento/para idade (cm)	125,0 ± 27,6	98,3 ± 28,0	0,000
Escore z de altura/comprimento/idade	–1,1 (–1,6 – –0,8)	–3,9 (–4,7 – –2,5)	0,001
IMC/idade	16,7 (15,5 – 19,6)	15,1 (4,3 – 16,9)	0,023
Escore z de IMC/idade	0,1 ± 1,4	–0,6 ± 1,4	0,094
Adequado (n, %)	14 (32,5)	16 (37,2)	0,114
Abaixo do peso	2 (4,6)	4 (9,3)	
Sobrepeso/obesidade	6 (13,9)	1 (2,3)	
Ureia (mg/dL)	78,7 ± 29,1	92,4 ± 39,6	0,204
Creatinina (mg/dL)	1,4 (1,0 – 1,9)	1,4 (0,9 – 1,8)	0,307
Estágio da DRC			
III	14	10	0,425
IV	7	8	
V	1	3	
TFG (mL/min/1,73m^2^)	34,6 ± 15,1	30,9 ± 13,5	0,411
Cálcio (mg/dL)	9,8 (9,5 – 10,2)	9,4 (8,8 – 10,0)	0,059
Fosfato (mg/dL)	5,0 ± 0,5	5,0 ± 1,5	0,862
Normal para a idade (n, %)	19 (44,1)	11 (25,5)	0,035
Elevado para a idade	3 (6,9)	7 (16,2)	
Baixo para a idade	0 (0,0)	3 (6,9)	
Fosfatase alcalina (U/L)	319 (230 – 399)	274 (207 – 563)	0,971
Normal para a idade (n, %)	9 (20,9)	7 (16,2)	0,607
Elevado para a idade	13 (30,2)	14 (32,5)	
Paratormônio (pg/mL)	138 (95 – 227)	132 (64 – 303)	0,789
Bicarbonato (mEq/L)	21 (20 – 23)	20 (19 – 22)	0,103
< 22mEq/L (n,%)	11(50,0)	15 (71,4)	0,151
Hemoglobina (g/dL)	11,0 ± 1,6	11,1 ± 2,2	0,239
Anemia (n, %)	2 (9,0)	6 (28,5)	0,101
Albumina (g/dL)	4,5 (4,3 – 4,6)	4,5 (4,1 – 4,8)	0,864
25OH vitamina D (ng/mL)	41 (34,2 – 44,2)	40 (30,5 – 47,5)	0,942

Nota – Os dados são expressos como (n, %), média e DP, ou mediana, intervalo de 25–75%,

* n = 36,

** n = 34.

Abreviações – TFG: taxa de filtração glomerular.

**Figura 1:** Correlação entre níveis iniciais de PTH e escore z de altura/comprimento.

Uma subanálise com crianças menores de cinco anos de idade ao final do período do estudo (n = 17) mostrou que 10 (58,8%) tiveram o crescimento prejudicado. Embora essas crianças apresentassem níveis mais baixos de creatinina (p = 0,032) e ureia (p = 0,015), elas demonstraram menor escore z de peso/idade (0,031) e menor escore z de IMC/idade (p = 0,04). Não foi observada diferença no fosfato sérico quando analisado por faixa etária (Tabela 4).

**Tabela 4:** Dados antropométricos e laboratoriais de crianças com cinco anos de idade ou menos, cujo crescimento melhorou e cujo crescimento foi mantido ou diminuiu

	Melhoria do crescimento n = 7	Manutenção ou redução do crescimento n = 10	P
Idade ao final do período do estudo (anos)	5 (2,0 – 5,0)	2 (1,7 – 2,5)	0,08
Sexo (n, %)			
Menino	5 (29,4)	6 (35,2)	0,62
Menina	2 (11,7)	4 (23,5)	
Acompanhamento (meses)	29 (21,0 – 60,0)	21 (9,0 – 33,7)	0,18
Altura/comprimento/idade (cm)	89,3 ± 10,5	79,9 ± 15,6	0,18
Escore z de altura/comprimento/idade	–2,9 ± 1,3	–3,2 ± 1,9	0,69
IMC/para idade	16,9 ± 1,6	15,2 ± 1,8	0,71
Escore z de IMC/idade	0,95 ± 1,1	–0,47 ± 1,6	0,04
Adequado (n, %)	5 (29,4)	7 (41,1)	0,33
Abaixo do peso	0 (0,0)	2 (11,7)	
Sobrepeso/obesidade	2 (11,7)	1 (5,8)	
Ureia (mg/dL)	115,0 ± 37,0	66,0 ± 35,6	0,01
Creatinina (mg/dL)	2,3 (1,4 – 3,2)	1,1 (0,8 – 1,6)	0,03
Estágio da DRC			
III	2	6	0,25
IV	2	3	
V	3	1	
Taxa de filtração glomerular (mL/min/1,73m^2^)	22,5 ± 14,1	38,0 ± 19,0	0,74
Bicarbonato (mEq/L)	20 (11 – 25)	21 (18 – 22)	0,08
< 22 mEq/L (n, %)	2 (11,7)	3 (17,6)	0,94
Hemoglobina (g/dL)	11,6 ± 2,0	10,5 ± 2,2	0,33
Anemia (n, %)	2 (11,7)	2 (11,7)	0,68
Cálcio (mg/dL)	9,4 ± 1,6	9,7 ± 0,8	0,67
Fosfato (mg/dL)	5,7 (5,7 – 6,7)	4,7 (4,3 – 5,7)	0,02
Normal para a idade (n, %)	5 (29,4)	7 (41,1)	0,66
Elevado para a idade	2 (11,7)	2 (11,7)	
Baixo para a idade	0 (0,0)	1(5,9)	
Fosfatase alcalina (U/L)	343 ± 207	362 ± 200	0,84
Normal para a idade (n, %)	2 (11,7)	5 (29,4)	0,37
Elevado para a idade	5 (29,4)	5 (29,4)	
Paratormônio (pg/mL)	120 (51 – 361)	125 (77 – 414)	0,92
Albumina (g/dL)	4,2 ± 0,5	4,2 ± 0,7	0,95
25OH vitamina D (ng/mL)	40,8 ± 14,7	43,5 ± 6,6	0,62

Nota – Os dados são expressos como (n, %), média e DP, ou mediana, intervalo de 25–75%.

## Discussão

O déficit de crescimento é uma das principais complicações da DRC em crianças. Cada unidade de redução no escore z de altura/comprimento para idade está associada a um aumento de 14% na mortalidade das crianças afetadas^8^. Em geral, a prevalência de baixa estatura em crianças com DRC varia de 30 a 50%^9^. O estudo PICCOLO MONDO com 225 crianças de cidades em quatro continentes, incluindo a cidade brasileira de Curitiba, constatou que 39% das crianças latino-americanas em hemodiálise tinham baixa estatura^9^. Em nossa série, verificamos que 51% das crianças apresentavam baixa estatura, que foi altamente prevalente em crianças mais jovens, incluindo o subconjunto de crianças abaixo de cinco anos. Esses dados contrastam com os resultados de estudos populacionais brasileiros, como a Pesquisa Nacional de Demografia e Saúde (PNDS) e a III Pesquisa Estadual de Saúde e Nutrição (PESN), que mostram uma prevalência de baixa estatura na população geral de crianças menores de cinco anos de idade de 7,0% no Brasil, 5,7% na região Nordeste e 8,7% no estado de Pernambuco^10^.

A desnutrição pode desempenhar um papel nesses cenários. No entanto, crianças com DRC apresentam um risco adicional de desnutrição uma vez que podem manter um elevado gasto energético em repouso ao mesmo tempo em que perdem massa magra e mantêm a massa de gordura corporal, levando à desnutrição proteico-energética (DPE) e resultando em comprometimento do crescimento. A recuperação do crescimento pode ser incompleta, mesmo nas crianças que recebem a ingestão ideal de calorias e proteínas^11^. Mesmo assim, a otimização da ingestão calórica em crianças com DRC é a estratégia mais eficaz para melhorar o crescimento^1^. É essencial assegurar a ingestão nutricional adequada e apropriada, principalmente em crianças pequenas. Um estudo recente mostrou benefícios no ganho de peso e altura/comprimento em crianças alimentadas por sonda enteral e considerou seu uso em crianças com DRC menores de seis anos de idade com baixo IMC^12^.

Além disso, condições específicas da DRC, como distúrbios minerais e ósseos, contribuem para a baixa estatura nessas crianças. O fosfato é conhecido como um “assassino silencioso” devido ao seu efeito nos distúrbios minerais e ósseos e à sua associação com calcificações vasculares. No entanto, a ingestão de fosfato está associada à ingestão de proteínas^13^. Nossos dados demonstraram que as crianças com baixa estatura apresentaram pior controle de fosfato do que suas contrapartes. Apesar de nossa rotina clínica de orientações dietéticas e medicamentos quelantes de fósforo, os pacientes apresentaram hiperfosfatemia, provavelmente devido à ingestão de proteínas e ao excesso de fosfato “escondido” nos aditivos alimentares. A abordagem nutricional da DRC tem como objetivo fornecer proteína suficiente para evitar a desnutrição e permitir o crescimento adequado, além de evitar hiper e hipofosfatemia. Nossos resultados ressaltam que a nutrição desses pacientes deve ser melhor monitorada, garantindo o fornecimento de proteínas e controlando a ingestão de fosfato.

Outro fator de risco para o retardo no crescimento é o hiperparatireoidismo secundário na DRC, uma vez que ele pode destruir a arquitetura óssea metafisária e resultar em interrupção do crescimento^14^. A DRC causa resistência óssea ao PTH, aumentando seus níveis nos estágios iniciais da doença^15^. Alguns autores argumentam que esse aumento é uma resposta adaptativa adequada ao declínio da função renal. Eles mostram uma associação positiva entre PTH e potencial de crescimento^16^. Outros pesquisadores sugerem que níveis mais elevados de PTH (até duas vezes o valor esperado para a idade) estão associados a um melhor crescimento nessas crianças^17^. Observamos níveis séricos elevados de PTH durante o acompanhamento do estudo. Quanto à fosfatase alcalina, 63% das crianças apresentaram níveis séricos elevados para sua idade durante o período do estudo. Além disso, observamos uma correlação inversa entre os níveis iniciais de PTH e o escore z de altura/idade avaliado no início e no final do período de acompanhamento. Esses resultados corroboram o diagnóstico de distúrbio mineral e ósseo da DRC e seu impacto negativo no crescimento de crianças com DRC.

A acidose metabólica é conhecida por degradar proteínas, aumentar a produção endógena de corticosteroides, reduzir a cartilagem de crescimento, reduzir a secreção do hormônio do crescimento (GH) e aumentar a resistência a ele^18^. Além disso, a acidose está associada à maior mortalidade em pacientes com DRC, demonstrando a importância do controle adequado. O estudo 4C (*Cardiovascular Comorbidity in Children with CKD*) em 704 crianças com DRC demonstrou uma prevalência de acidose metabólica em 43%, 60% e 45% na DRC 3, 4 e 5, respectivamente, e níveis de bicarbonato abaixo de 18 mEq/L não foram associados ao crescimento^19^. Nossa análise não mostrou nenhuma associação entre níveis de bicarbonato e crescimento. Apesar desses resultados, a acidose metabólica esteve presente em 74% dos pacientes no início do estudo e em 60% ao final do acompanhamento. Em nossa prática, prescrevemos bicarbonato de sódio. Entretanto, muitos de nossos pacientes têm dificuldade de acesso a medicamentos e não aderem ao tratamento. Além disso, há relatos de que o comprometimento médico afeta o crescimento de crianças com DRC^20^. Esses dados reforçam a necessidade de desenvolver estratégias para melhorar a adesão desses pacientes e a vigilância rigorosa para manter níveis adequados desse íon.

Outro fator que afeta o crescimento de crianças com DRC é a anemia. Quarenta e nove por cento de nossos pacientes apresentou anemia no início do acompanhamento. Observamos uma melhora nos níveis de hemoglobina ao final do período do estudo, provavelmente devido ao tratamento. No entanto, a anemia persistiu em 18% dos casos. O relatório do *North American Pediatric Renal Transplant Cooperative Studies* (NAPRTCS) mostrou que 46% das crianças apresentaram anemia no início do estudo, o que esteve associado ao retardo do crescimento. Boehm et al.^21^ observaram uma melhora no crescimento em apenas 40% das crianças sob tratamento com eritropoietina. Esse fato nos chama a atenção para a necessidade de melhorar a vigilância e a adequação do tratamento da anemia. Ao mesmo tempo, também pode refletir a evolução da própria DRC.

A associação entre baixo peso ao nascer, prematuridade e baixa estatura em crianças com DRC já é conhecida^22^. Greenbaum et al.^23^ mostraram baixo peso ao nascer em 17% das crianças com DRC, o que foi semelhante ao nosso achado (n = 5; 14,7%). Entretanto, não encontramos uma associação com estatura.

O estágio da DRC pode afetar o crescimento, e crianças nos estágios iniciais da doença têm maior probabilidade de apresentar estatura adequada. Os fatores implicados na patogênese e na progressão da DRC podem estar envolvidos no déficit de crescimento nesses casos^24^. Além da função renal, o tempo de doença pode afetar o crescimento. Ingulli e Mak^25^ observaram que crianças com DRC apresentavam retardo progressivo no crescimento. Semelhante a esse estudo, nossos resultados mostraram uma piora no escore z de altura das crianças (–1,89 ± 1,84 e –2,4 ± 1,67; p = 0,011) durante o acompanhamento de 36 meses.

Aqui, chamamos a atenção para as características da população estudada. Faz parte de nossa prática diária (dados não exibidos) atender crianças de famílias de baixa renda que chegam ao atendimento em um estágio avançado da doença, vêm de cidades distantes do centro e, portanto, têm dificuldade em manter um acompanhamento regular. É fundamental destacar o impacto das condições socioeconômicas nos desfechos da DRC^26^. O baixo nível socioeconômico das famílias implica em falta de conhecimento sobre a doença, baixa adesão e falta de recursos para a compra de medicamentos, o que é um problema comum em nosso país^27^.

O efeito positivo do uso de GH na altura final de crianças com DRC já está bem documentado^28^. As diretrizes europeias mais recentes recomendam a terapia com GH em crianças de baixa estatura com DRC estágio 3–5D, sugerindo a modificação dos fatores de risco antes de seu uso^29^. Infelizmente, apesar dos benefícios do GH, ele ainda é pouco utilizado em crianças com DRC. Uma situação semelhante ocorreu em nossa população, onde nenhum paciente foi submetido à terapia com GH recombinante. Esse cenário preocupante mostra a importância da implementação de uma política de acesso mais eficaz a essa terapia.

As intervenções precoces são essenciais e muito mais eficazes se forem iniciadas antes da diálise. Haffner^30^ recentemente publicou estratégias para orientar a prevenção e tratamento do retardo de crescimento nessas crianças com DRC. As diretrizes sugerem focar na preservação da função renal por meio da normalização da pressão arterial e da proteinúria com o uso de inibidores do sistema renina-angiotensina-aldosterona, tratar a anemia com estimuladores de eritropoiese, cuidar da adequação energética, incluindo o uso de sonda enteral ou gastrostomia quando necessário, corrigir distúrbios hidroeletrolíticos e ácido-básicos, controlar os níveis de PTH na faixa-alvo da DRC, usar GH recombinante quando indicado e transplante renal precoce com protocolos de imunossupressão com doses mínimas de esteroides^30^.

As limitações de nosso estudo foram sua natureza retrospectiva, de centro único e o pequeno tamanho da amostra, o que nos restringiu a expandir as análises. No entanto, o estudo revelou elevada prevalência de baixa estatura, que persistiu durante o acompanhamento, especialmente em crianças pequenas. Além disso, os níveis de PTH foram inversamente correlacionados com o escore z de altura/idade, e a deficiência de crescimento nessas crianças foi associada a um pior estado nutricional. Nossos resultados reforçam a importância de monitorar o crescimento dessas crianças, manter o controle do hiperparatireoidismo e fornecer suporte nutricional.
